# Evaluation of commercial DNA and RNA extraction methods for high-throughput sequencing of FFPE samples

**DOI:** 10.1371/journal.pone.0197456

**Published:** 2018-05-17

**Authors:** Stine H. Kresse, Heidi M. Namløs, Susanne Lorenz, Jeanne-Marie Berner, Ola Myklebost, Bodil Bjerkehagen, Leonardo A. Meza-Zepeda

**Affiliations:** 1 Department of Tumor Biology, Norwegian Radium Hospital, Oslo University Hospital, Oslo, Norway; 2 Genomics Core Facility, Department of Core Facilities, Norwegian Radium Hospital, Oslo University Hospital, Oslo, Norway; 3 Department of Pathology, Norwegian Radium Hospital, Oslo University Hospital, Oslo, Norway; 4 Department of Clinical Science, University of Bergen, Bergen, Norway; 5 Norwegian Cancer Genomics Consortium (cancergenomics.no), Norwegian Radium Hospital, Oslo University Hospital, Oslo, Norway; University of Helsinki, FINLAND

## Abstract

Nucleic acid material of adequate quality is crucial for successful high-throughput sequencing (HTS) analysis. DNA and RNA isolated from archival FFPE material are frequently degraded and not readily amplifiable due to chemical damage introduced during fixation. To identify optimal nucleic acid extraction kits, DNA and RNA quantity, quality and performance in HTS applications were evaluated. DNA and RNA were isolated from five sarcoma archival FFPE blocks, using eight extraction protocols from seven kits from three different commercial vendors. For DNA extraction, the truXTRAC FFPE DNA kit from Covaris gave higher yields and better amplifiable DNA, but all protocols gave comparable HTS library yields using Agilent SureSelect XT and performed well in downstream variant calling. For RNA extraction, all protocols gave comparable yields and amplifiable RNA. However, for fusion gene detection using the Archer FusionPlex Sarcoma Assay, the truXTRAC FFPE RNA kit from Covaris and Agencourt FormaPure kit from Beckman Coulter showed the highest percentage of unique read-pairs, providing higher complexity of HTS data and more frequent detection of recurrent fusion genes. truXTRAC simultaneous DNA and RNA extraction gave similar outputs as individual protocols. These findings show that although successful HTS libraries could be generated in most cases, the different protocols gave variable quantity and quality for FFPE nucleic acid extraction. Selecting the optimal procedure is highly valuable and may generate results in borderline quality specimens.

## Introduction

Large archives of formalin-fixed paraffin-embedded (FFPE) tissues are held in clinical laboratories, representing an invaluable source of material for biomedical research. FFPE blocks enable prolonged storage of clinical samples such as tumour biopsies and operation material, preserving tissue morphology for pathological examination and nucleic acids for molecular analysis. However, as we enter the era of molecular pathology and personalized medicine, the molecular preservation and quality of proteins and nucleic acids become critical. The ability to reliably investigate nucleic acids from FFPE material is thus important, and should also be taken into account in tissue fixation procedures.

In general, nucleic acid extraction from FFPE samples yields inferior quality material compared to extraction from fresh frozen-tissue, and detecting low frequency genetic variants from FFPE samples poses significant challenges. However, high concordance has been reported between paired fresh frozen and FFPE samples in molecular analyses [1]. The nucleic acid quality and integrity are affected by the pH of the fixative, the length of tissue fixation, the age and storage condition of tissue blocks and the extraction method used [2–4]. Formaldehyde, the active component of formalin, chemically cross-links the nucleic acids with the surrounding proteins and may also modify the nucleotides. Consequently, this leads to fragmentation of nucleic acids. In addition, enzymatic reactions, such as those used in reverse transcription and PCR amplification may be inhibited. Furthermore, non-reproducible sequence artefacts are introduced through deamination of cytosine to uracil, which can be misinterpreted as mutations [5, 6].

Robust DNA and RNA extraction protocols are required for accurate identification and quantification of somatic variants in FFPE tissue specimens, and this will improve both research and routine clinical management of cancer. Multiple analyses are routinely performed on the same FFPE sample, such as immunohistochemistry (IHC), *in situ* hybridization (ISH), Sanger sequencing and PCR experiments. In addition, variant detection based on DNA or RNA sequencing is becoming increasingly important for diagnostics and therapeutic decision making, and high-throughput sequencing (HTS) is now being implemented into routine molecular pathology.

Considerable efforts have been undertaken to optimize methods for extraction of high-quality DNA from FFPE samples, and several commercial extraction protocols have been developed for this specific purpose [7]. In the majority of these protocols, the digestion of cross-linked proteins and solvation of FFPE tissue is performed in a tissue lysis buffer containing proteinase K. Deparaffinization of sections is usually done using xylene or other less toxic organic solvents for solubilisation and phase separation. Paraffin can also be dissociated using lysis buffer, alternatively combined with specific techniques like adaptive focused acoustics (AFA), simultaneously rehydrating the tissue. A reverse formalin cross-linking step to increase DNA and RNA performance in downstream applications is also frequently performed. The current purification of DNA or RNA molecules is mainly based on silica adsorption or paramagnetic bead based binding technologies [7].

Various studies have addressed the crucial choice of the DNA and RNA extraction method, its impact on nucleic acid integrity and downstream performance. These cover both manual and automatic extraction systems for DNA or RNA, evaluating amplifiability using PCR or HTS [1, 8–12]. Large variations were observed both when it comes to quantity and quality. Simultaneous extraction of both amplifiable DNA and RNA has also been described [1, 13, 14]. In many cases, the amount of FFPE material available is limited, and it can be difficult to obtain material for several analyses, like determining both DNA variants and RNA fusions (rearrangements).

In this study, we have performed an extensive comparison of different nucleic acid extraction protocols for FFPE material, using eight different extraction protocols from seven kits from three different commercial vendors. This included two protocols where both DNA and RNA were obtained simultaneously that have not previously been compared in other studies. In addition to quantity and quality of the nucleic acids extracted, performance on HTS applications using Agilent SureSelect XT (Agilent Technologies, Santa Clara, CA, US) for variant detection in DNA and Archer FusionPlex Sarcoma Assay (ArcherDX, Boulder, CO, USA) for fusion detection in RNA were evaluated.

## Materials and methods

### FFPE material and pathological examination

Archival FFPE blocks from five sarcoma samples were selected from the biobank at the Department of Pathology, Oslo University Hospital. Preparation of the FFPE blocks was performed following standard procedures at the Department of Pathology. Varying levels of cellularity, necrosis, tumour content and tissue size were present among the five sarcoma samples analysed. All tumours were diagnosed according to the World Health Organization (WHO) classification [15], and clinical information was retrieved from a MEDinsight database at Oslo University Hospital. For two of the samples, fusion genes were detected using RT-PCR according to standard molecular testing at the Department of Pathology. Data for all FFPE blocks are presented in Table 1.

**Table 1:** Data for FFPE blocks.

Sample	Sarcoma subtype	Cellularity	Necrosis (%)	Tissue size (mm x mm)	Tumour size (mm x mm)	Age FFPE block (years)	Fusion gene RT-PCR
**SARC1**	Alveolar soft tissue sarcoma	Cell rich	50	28x23	18x16	4	Not done
**SARC2**	Alveolar rhabdomyosarcoma	Moderate	<5	15x15, multiple pieces	Diffuse growth, 30% of the area	5	Not done
**SARC3**	Synovial sarcoma	Cell rich	40	18x15	17x15	3	SS18-SSX1/2/4
**SARC4**	Extraskeletal myxoid chondrosarcoma	Moderate	<5	25x18	18x16	2	EWSR1-NR4A3*
**SARC5**	Ewing sarcoma	Moderate	<5	20x20	14x7	2	EWSR1-FLI1

*Detected by HTS

### DNA and RNA extraction methods

DNA and RNA were extracted from FFPE sections using eight different protocols from seven kits from three different commercial vendors (Table 2). For DNA, the AllPrep DNA/RNA FFPE Kit, GeneRead DNA FFPE Kit and QIAamp DNA FFPE Tissue Kit from QIAGEN and truXTRAC FFPE DNA Kit from Covaris were used. For RNA, the AllPrep DNA/RNA FFPE Kit and RNeasy FFPE Kit from QIAGEN, Agencourt FormaPure Kit from Beckman Coulter and truXTRAC FFPE RNA Kit from Covaris were used. For the AllPrep DNA/RNA FFPE Kit, simultaneous extraction of DNA and RNA was done. For the truXTRAC FFPE DNA and RNA kits, both separate and simultaneous extraction of DNA and RNA were done.

**Table 2:** Overview of nucleic acids extracted with the different extraction kits from commercial vendors.

Extraction kit	Nucleic acid extracted	Vendor
AllPrep DNA/RNA FFPE Kit	DNA and RNA	QIAGEN, Inc., Hilden, Germany
GeneRead DNA FFPE Kit	DNA	QIAGEN, Inc., Hilden, Germany
QIAamp DNA FFPE Tissue Kit	DNA	QIAGEN, Inc., Hilden, Germany
truXTRAC FFPE DNA Kit	DNA/DNA and RNA*	Covaris, Inc., Woburn, MA, USA
Agencourt FormaPure Kit	RNA	Beckman Coulter, Inc., Indianapolis, IN, USA
RNeasy FFPE Kit	RNA	QIAGEN, Inc., Hilden, Germany
truXTRAC FFPE RNA Kit	RNA/DNA and RNA*	Covaris, Inc., Woburn, MA, USA

*The simultaneous extraction of DNA and RNA uses both truXTRAC kits

DNA and RNA extractions were performed three times per kit, except for the AllPrep DNA/RNA FFPE, FormaPure and truXTRAC FFPE DNA and RNA kits, where extraction was performed twice due to limited material. DNA and RNA were extracted from a single 10 μm FFPE section from the five sarcoma samples, using a total of 95 sections. Equal amount of starting material was used for all methods of extraction, and consecutive FFPE sections for each block were randomized before extraction. DNA and RNA were extracted as soon as possible after sectioning in order to prevent oxidation of the nucleic acids.

The DNA extractions were done following the manufacturers’ instructions. All samples were treated with RNAse and the DNA was eluted in a volume of 100 μl. The RNA extractions were done following the manufacturers’ instructions, with some exceptions for the AllPrep and FormaPure kits, where specific recommendations from ArcherDX were followed since the Archer FusionPlex Sarcoma Assay from ArcherDX was used for HTS analysis of the RNA samples. For AllPrep, the protocol for extraction of total RNA that does not include small RNAs was followed, deparaffinization was performed using Deparaffinization Solution (QIAGEN), and the RNA was eluted in 30 μl water. For FormaPure, the samples were digested with proteinase K overnight instead of 60 minutes, and the RNA was eluted in 40 μl water. For the other methods, the RNA was eluted in a volume of 30 μl. For all RNA protocols, no DNAse treatment was performed.

### DNA and RNA yield, integrity and amplifiability

DNA and RNA yield were measured using the Qubit dsDNA BR Assay Kit and RNA BR/HS Assay Kits, respectively, and the Qubit 3.0 Fluorometer from Life Technologies. DNA integrity (DIN) values were determined using the 2200 TapeStation system with the D1000 ScreenTape. RNA integrity (RIN) values were determined using the 2100 Bioanalyzer system with the RNA 6000 Nano Kit (Agilent Technologies, Santa Clara, CA, USA).

DNA amplifiability was measured using the FFPE QC kit from Illumina (Illumina Inc., San Diego, CA, USA) according to the manufacturer’s instructions using 4 ng of FFPE DNA input. RNA amplifiability was measured using the PreSeq QC assay from ArcherDX according to the manufacturer’s instructions. 50 ng of RNA was used as input whenever possible, but for some samples all RNA amount available (< 50 ng) was used. Real-time qPCR was performed using the Applied Biosystems 7900HT Fast Real-Time PCR system (Thermo Fisher Scientific). The QC Template Reagent and the Universal RNA from Ambion (Thermo Fisher Scientific) were run in parallel to serve as a control sample for DNA and RNA, respectively. Undetected samples were censored to Ct 45. For each sample, a delta Ct value was computed by subtracting the average Ct value for the QC Template from the average Ct value for each sample.

### DNA high-throughput sequencing

For DNA HTS library preparations, DNA was selected from a parallel of extraction giving average yields, for all the different methods. For the truXTRAC extraction method, DNA simultaneously extracted with RNA was used. Libraries for DNA HTS were prepared from 1 μg DNA following the SureSelect XT protocol (Agilent Technologies) with a custom SureSelect in-solution 900 cancer genes capture panel developed by the Norwegian Cancer Genomics Consortium (cancergenomics.no) (Namlos, Zaikova et al. 2017). The libraries were sequenced paired-end (2 x 151 bp) on a HiSeq4000 instrument (Illumina Inc., San Diego, CA, US) using Sequencing by Synthesis chemistry.

BCL files generated by the Illumina sequencing system were processed using the Illumina Bcl2fastq (v. 2.20) software to generate and demultiplex fastq files. The mapping, alignment, quality assessment and somatic variant calling were performed as previously described (Namlos, Zaikova et al. 2017). A Randomized downsize of the data based on coverage per sample was done to compare variant calling for all DNA extraction methods. Variant calling was done with MuTect [16] and Strelka [17], using an artificial control as normal. The variants were annotated and filtered in Variant Studio v2.2.1 (Illumina) using a normal variation population filter (< 1% ExAC database), keeping exonic variants with allele frequency > 5% and coverage > 100x. In addition, a randomized downsize of the data based on number of reads per sample was done to examine the complexity of the different DNA libraries. Access to the HTS data is provided upon request.

### RNA high-throughput sequencing

For RNA HTS library preparations, RNA was selected from a parallel of extraction giving average yields, for all the different methods. For the truXTRAC extraction method, RNA simultaneously extracted with DNA was used. Libraries for RNA HTS were made using the Archer FusionPlex Sarcoma Assay and the Archer Universal RNA Reagent Kit v2 for Illumina (ArcherDX), following the manufacturer’s instructions. For samples SARC3 and SARC4, 200 ng RNA was used as input, and all available amount of RNA (20–160 ng) was used for SARC5. In brief, cDNA was first synthesized from the RNA using random priming, then end repair, dA-tailing and adapter ligation were performed, using the molecular barcode (MBC) adapters A13-24 for Illumina (ArcherDx). Then two rounds of PCR with gene-specific primers from the Archer FusionPlex Sarcoma Assay were performed. The final libraries were quantified using the KAPA Biosystems qPCR Kit for Illumina (KAPA Biosystems, Wilmington, MA, USA), assuming a 250 bp fragment length. Barcoded libraries were pooled at equimolar concentrations and sequenced using the MiSeq Sequencing System from Illumina with the MiSeq v2 300 cycle reagent kit (Illumina).

Fastq files from MiSeq were analysed using the Archer Analysis 5 bioinformatics platform from ArcherDX using default settings for the FusionPlex Sarcoma Assay. The fusion detection algorithm relies on the specificity of the gene specific primers used in the amplification steps in the anchored multiplex PCR (AMP) process. The software requires a single read spanning two separate genes to be considered a fusion candidate. Archer Analysis performs all secondary analysis, and many tertiary analysis steps with annotations from the Archer Quiver Fusion Database, as well as sources such as Ensembl VEP, Clinvar and COSMIC. In addition, a randomized downsize of the total number of reads for each sarcoma sample across extraction methods was done to be able to compare all the methods under similar number of reads and to assess the impact of read depth on fusion detection. The down-sampling was done according to the sample with lowest total number of reads (> 1.5 million reads). Access to the HTS data is provided upon request.

### Statistical analysis

Statistical analysis was performed to determine if there was a significant difference in DNA and RNA yield, DIN and RIN values, DNA and RNA amplifiability and yield of DNA HTS libraries and QC metrics for RNA HTS libraries, obtained using the different extraction methods. A Wilcoxon Signed Ranks test was performed taking the median value of each sample when two or three replicates were available, and testing the methods against one and each other.

### Ethics statement

The project (S-06133) was approved by the Regional Ethical Committee for Southern Norway, and informed, written consent was obtained from the patients.

## Results and discussion

### DNA and RNA yield

DNA and RNA were extracted from the same archival FFPE blocks from five sarcoma samples using different extraction kits from commercial vendors in order to determine the best extraction method for downstream HTS applications. The QIAamp DNA FFPE Tissue Kit, miRNeasy FFPE Kit and AllPrep DNA/RNA FFPE Kit from QIAGEN have been shown to perform well in comparison with other DNA and RNA protocols in previous studies [1, 12, 14]. However, comparisons with the other kits evaluated in this study have not been published so far.

**Fig 1:**
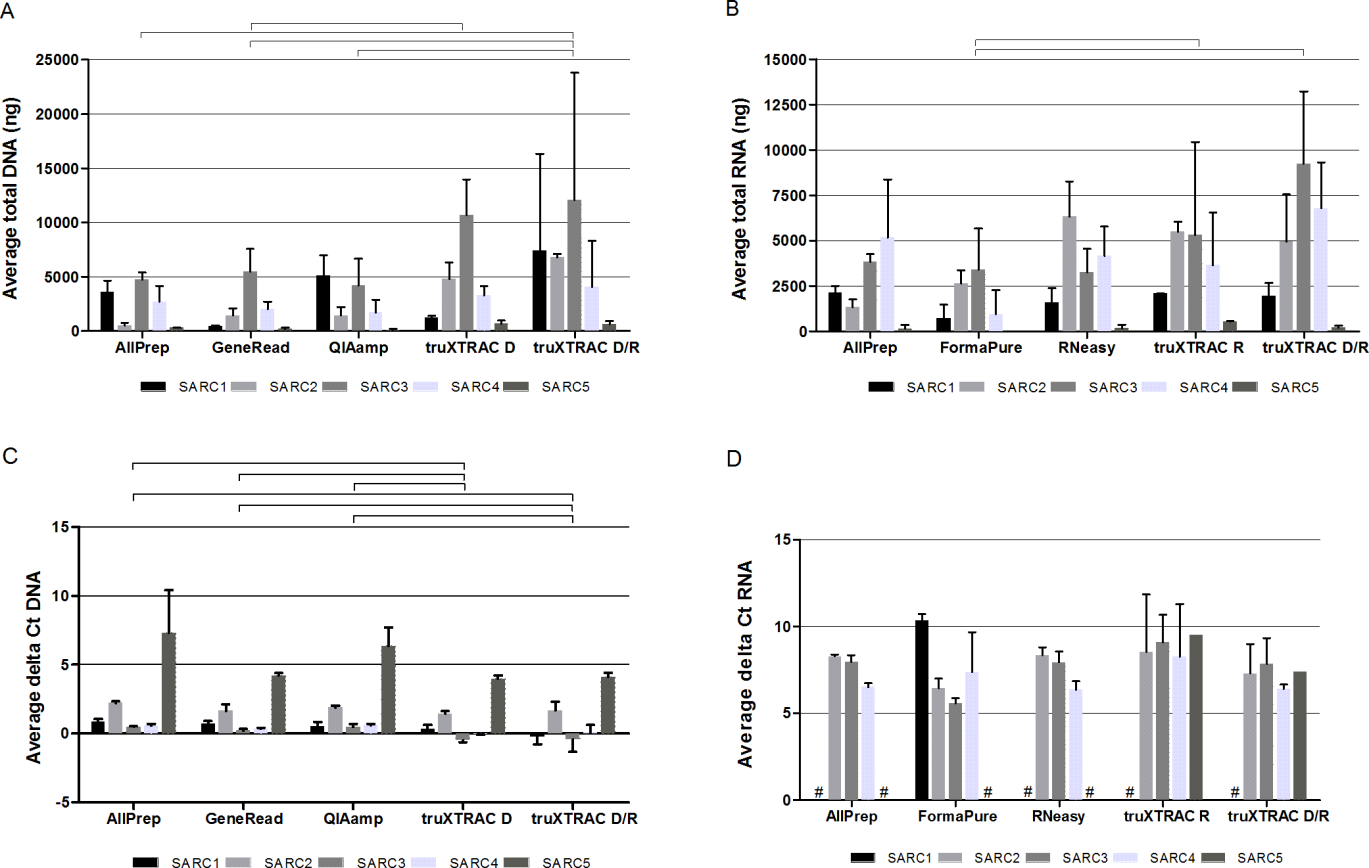
Yield and amplifiability of extracted DNA and RNA. (A) Average total amount of DNA. (B) Average total amount of RNA. (C) Amplifiable DNA quantified with the FFPE QC kit from Illumina. (D) Amplifiable RNA quantified with the PreSeq QC assay from ArcherDx. The average total amount and average delta Ct values for the different samples and extraction methods are shown. The standard deviation is shown as vertical bars. Methods with significant differences in yield are marked as connected with horizontal bars (p < 0.05). Samples with Ct values > 45 were censored giving a delta Ct of 21, shown as #.

Equal amount of starting material and a randomized order of the consecutive FFPE block sections were used for all methods of extraction. The extracted DNA and RNA were quantified using fluorescent dye-based quantification, and the average total amount for the different samples and extraction methods are shown in Fig 1A and 1B and S1 Table. For DNA, the highest yields were in general obtained using the truXTRAC kit, although there were variations between the samples and the individual extractions. The protocol with simultaneous extraction of both DNA and RNA gave higher DNA yields than the protocol with extraction of only DNA for the truXTRAC kit. The truXTRAC kit DNA/RNA (simultaneous protocol) gave significantly higher DNA yields compared to the AllPrep, GeneRead and QIAamp kits (all p = 0.043), whereas the truXTRAC kit (DNA only) gave significantly higher DNA yield compared to the GeneRead kit (p = 0.043). No significant differences in DNA yield were observed comparing the three QIAGEN kits. There were differences in the DNA amount obtained for the different samples, sample SARC3 gave the highest yield of DNA, whereas SARC5 gave a very low yield compared to the other samples, indicating a difference in quality of the FFPE blocks. Sample SARC1 and SARC3 were found to have >40% necrosis, but being particularly cell rich, these samples still contained sufficient nucleic acids for analysis. Sample SARC2 and SARC5 had the smallest tissue size (Table 1).

For RNA, the yields from the different extraction methods were more similar, although the FormaPure kit gave slightly lower RNA yields than the other extraction methods. Both protocols using the truXTRAC kit gave significantly higher RNA yields compared to the FormaPure kit (p = 0.043). As also seen for the DNA amounts, sample SARC5 gave by far a lower RNA yield than the other samples.

### DNA and RNA integrity and amplifiability

Nucleic acid integrity, including both fragment length and absence of chemical modifications, is important for successful amplification. The fragment length distributions for the extracted DNA and RNA obtained using the different extraction protocols are shown in S1 Fig, and the DIN and RIN values are shown in S1 Table. Taking all the samples into account, the truXTRAC DNA/RNA kit (simultaneous protocol) gave significantly higher DIN values (p = 0.04) than all methods but the AllPrep kit, whereas lowest integrity was obtained using the GeneRead kit. For RNA, the truXTRAC RNA kit (RNA protocol only) gave the highest RIN values, significantly higher than the RNeasy and FormaPure kits (p = 0.04). Smaller differences were seen for samples of high or very low nucleic acid integrity, but the choice of protocol was more important when having intermediate quality.

In order to determine the amplifiability of the extracted DNA and RNA obtained using the different extraction protocols, qPCR was performed using the FFPE QC kit and PreSeq QC assay, respectively. The average delta Ct values for the different samples and extraction methods are shown in Fig 1C and 1D and S1 Table. In line with the above results, the truXTRAC DNA/RNA kit (simultaneous protocol) gave significantly lower delta Ct values for DNA compared to the AllPrep, GeneRead and QIAamp kits (all p = 0.04), showing better amplifiability. Although equal amounts of DNA was used as input for the FFPE QC assay, in general there seemed to be a trend that samples with higher amounts of DNA extracted gave lower delta Ct values and opposite. Thus, the truXTRAC DNA/RNA kit (simultaneous protocol) did not only give significantly higher yield of DNA than the kits from QIAGEN, but also DNA of better quality.

For RNA, the PreSeq QC quality score takes both concentration, length and crosslinking into consideration. For sample SARC5, the extractions used for sequencing library preparation were not included in the PreSeq QC quantification due to limited amounts of RNA. No significant differences in delta Ct values, reflecting the amplifiability, were observed between the methods. In contrast to DNA, high yields of extracted RNA did not necessarily correspond to low delta Ct values. For sample SARC1, RNA extracted using the FormaPure kit was the only RNA to obtain a delta Ct value although the yield obtained was by far lower than with the other extraction methods.

### Detection of variants in DNA samples

Libraries for DNA HTS were made for four of the samples, SARC1-4, using Agilent SureSelect XT with a custom SureSelect in-solution 900 cancer gene panel. The quality and yield of SARC5 DNA were considered too low for library preparation. Two of the samples, SARC1 extracted using the GeneRead kit and SARC2 extracted using the AllPrep kit, did not have enough library yield to proceed to capturing. Thus, for two of the samples, DNA HTS was performed on libraries representing only three DNA extraction methods, while all four methods were included for the remaining two samples. No significant differences were seen between the methods for DNA library yield, neither for the libraries before capturing nor the final libraries, although the QIAGEN methods showed a trend towards more yield (Tables 3 and S1).

**Table 3:** Yield of final DNA HTS libraries obtained using DNA extracted with different extraction kits.

Total amount (nM)	AllPrep	GeneRead	QIAamp	truXTRAC DNA/RNA
**SARC1**	6.4	0.3*	4.7	2.7
**SARC2**	0.7*	4.6	5.1	3.3
**SARC3**	12.9	9.0	9.3	7.8
**SARC4**	5.9	6.4	9.8	3.5

The quantification was done using qPCR.

*These libraries were not sequenced due to too low yield.

**Fig 2:**
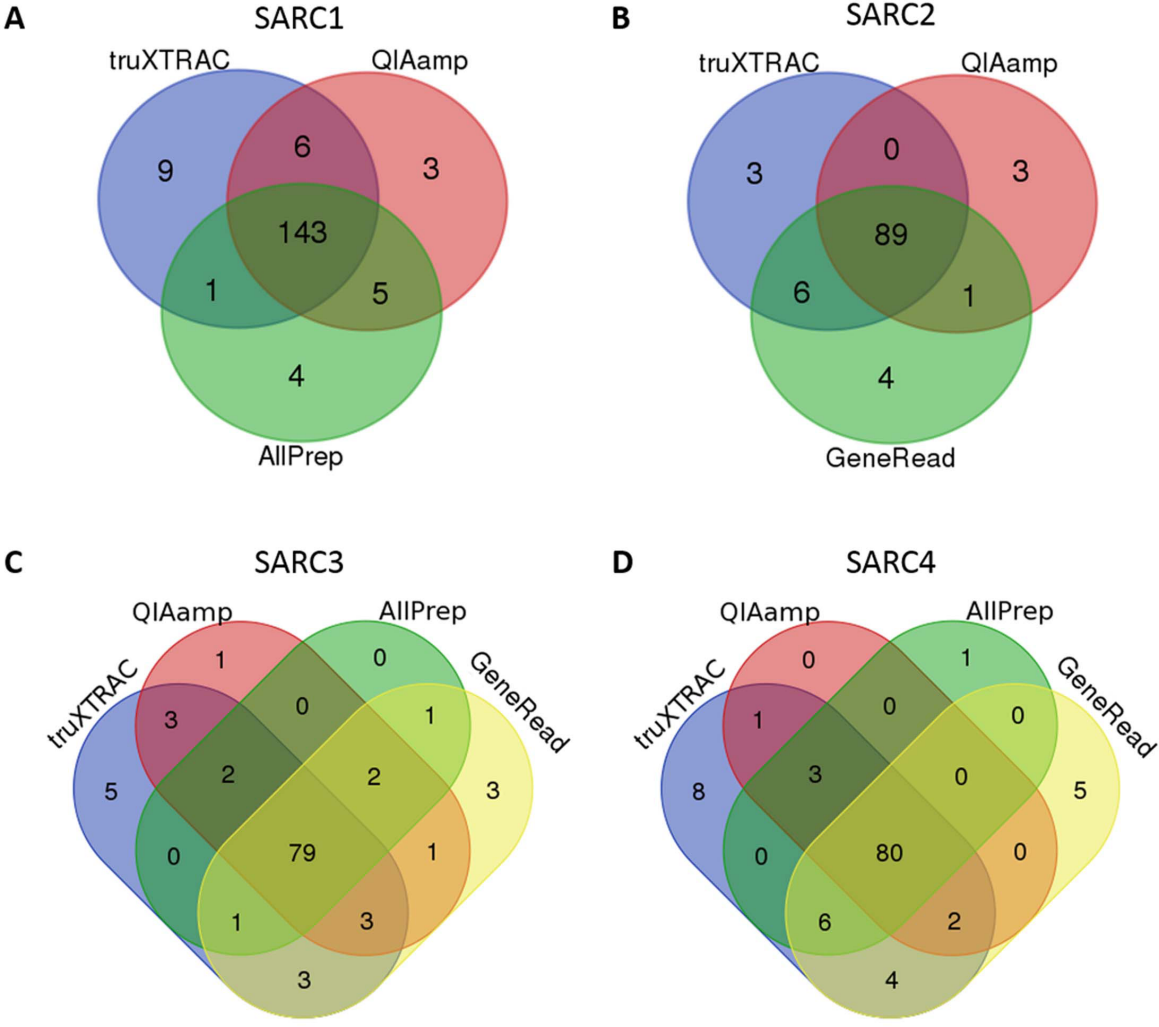
Variants identified in the samples extracted with different DNA extraction methods. (A-D) Venn diagram showing the distribution of variants between DNA extraction methods in four different FFPE samples, SARC1-4. Variants were detected by MuTect and Strelka, using an artificial control as normal. The data was filtered on exonic variants with allele frequency > 5% and coverage > 100x, being present at < 1% in the normal population (ExAC database).

To better compare the suitability of the various sequenced libraries for somatic variant calling, a randomized downsize of the data was done to be able to compare all the methods under similar coverage per sample, giving a mean coverage of 245-320x (S1 Table). The somatic variants identified for the different extraction methods and samples are shown in Fig 2, and the quality metrics for the sequencing are given in S1 Table. Overall, the majority of the variants called by MuTect and Strelka were concordant among the different extraction methods within a given sample.

In order to compare the complexity of the different libraries, we downsized the number of reads and calculated the duplication rates. No significant differences were seen between the extraction methods, although the libraries generated with AllPrep in general showed a lower duplication rate, indicating a more diverse library (Table 4).

**Table 4:** Complexity of final DNA HTS libraries represented by duplication rates of downsized libraries.

Total amount (nM)	AllPrep	GeneRead	QIAamp	truXTRAC DNA/RNA
**SARC1**	11.9	NA*	35.8	49.2
**SARC2**	NA*	32.6	36.9	29.0
**SARC3**	12.4	10.4	24.3	10.1
**SARC4**	19.6	19.6	23.8	41.9

*These libraries were not sequenced due to too low yield.

### Detection of fusion genes in RNA samples

For the three samples with known fusion genes, SARC3-5, libraries for RNA HTS were made using the Archer FusionPlex Sarcoma Assay. The library yields are given in Tables 5 and S1. Although there were no significant differences, libraries made from RNA extracted from the FormaPure and truXTRAC DNA/RNA kits (simultaneous extraction) showed a trend towards more yield. The library yield was by far the lowest for sample SARC5, reflecting the low quality of this sample.

**Table 5:** Yield of Archer FusionPlex Sarcoma Assay libraries obtained using RNA extracted with different extraction kits.

Total amount (nM)	AllPrep	FormaPure	RNeasy	truXTRAC R/D
**SARC3**	246.6	325.7	259.9	304.4
**SARC4**	348.9	316.0	312.7	355.9
**SARC5**	4.8	25.6	2.4	17.7

The quantification was done using the KAPA Biosystems qPCR Kit for Illumina.

Fusion detection was performed using the Archer Analysis 5 bioinformatics platform using default settings for the FusionPlex Sarcoma Assay (Tables 6 and S1). The quality metrics for the sequencing are given in S1 Table. A EWSR1-NR4A3 fusion was detected in RNA extracted with all methods for sample SARC4. The low quality of sample SARC5 was also reflected in the analysis, as the pathognomonic EWSR1-FLI1 fusion was not detected, which was also expected based on the obtained quantity and quality. Thus, for low quality FFPE samples, the choice of extraction method did not have any effect on the ability to detect fusions. For sample SARC3, a SS18-SSX2 fusion was detected with RNA extracted using the FormaPure and truXTRAC kits, but not with the AllPrep and RNeasy kits. For the two extractions where the fusion was not detected, the libraries were of poorer quality as shown by the low percentage of unique fragments providing less complex libraries, and the HTS data did not pass the Archer QC filter (S1 Table), reflecting a low quality of the extracted RNA.

**Table 6:** Fusion detection in Archer FusionPlex Sarcoma Assay libraries with total number of unique reads and down-sampled number of reads for each sarcoma sample separately.

Sample	Fusion	AllPrep Full/Down-sampled		FormaPure Full/Down-sampled		RNeasy Full/Down-sampled		truXTRAC R/D Full/Down-sampled	
**SARC3**	SS18-SSX2	No 62k (4.5)	No 62k (4.5)	Yes 141k (9.0)	Yes 127k (9.5)	No 70k* (4.7)	No 70k (4.7)	Yes 187k (10.1)	Yes 128k (9.9)
**SARC4**	EWSR1-NR4A3	Yes 190k (10.2)	Yes 153k (11.0)	Yes 184k (10.8)	Yes 152k (11.4)	Yes 109k* (7.9)	Yes 109k (7.9)	Yes 175k (8.7)	Yes 132k (9.5)
**SARC5**	EWSR1-FLI1	No 15.4k (1.1)	No 13.7k (1.2)	No 6.5k* (0.6)	No 6.5k (0.6)	No 5.8k (1.4)	No 5.8k (1.4)	No 40k (0.6)	No 15.6k (1.3)

Yes–fusion detected, No–fusion not detected. The total number and percentage (%) of unique fragments identified in libraries are given. The down-sampling was done according to the sample with lowest total number of reads (> 1.5 million reads) (labelled with *). See S1 Table for more detailed information.

To better compare the fusion detection capability of the different extraction methods, the number of reads per sample was normalized by down-sampling according to the sample with the lowest number of reads (> 1.5 million reads) for each sarcoma sample separately. The fusion gene analysis was then re-run, giving the same results for fusion detection as when all reads were included (Tables 5 and S1). Hence, the reasons for not detecting fusions in some of the samples were not related to the library read size. RNA extracted from the FormaPure kit gave the highest percentage of unique reads and on target reads, followed by the truXTRAC kit, although the difference was not significant compared to the other methods (Tables 5 and S1). This highlights the importance of library complexity and thus RNA quality for detection of somatic changes.

## Conclusions

The different DNA and RNA extraction methods examined in this study gave variable quantity, quality and performance of the isolated nucleic acids. For DNA extraction, the truXTRAC FFPE DNA kit from Covaris gave in general higher yield and more intact DNA fragments, however, no significant differences were observed in the quantity of libraries, DNA sequencing or variant calling among the different extraction methods tested. For detection of RNA transcripts from fusion genes, the truXTRAC FFPE RNA kit from Covaris and FormaPure kit from Beckman Coulter performed better identifying a fusion that was not detected with the kits from QIAGEN. The FormaPure and truXTRAC kits also had the highest percentage of unique read-pairs identified, providing a higher complexity of the HTS libraries and data. For both DNA and RNA, the truXTRAC simultaneous DNA and RNA extraction protocol performed just as well or even better than the separate truXTRAC protocols. In general, the choice of extraction method seems to be more crucial for RNA than for DNA. However, the findings show that selecting the optimal procedure for nucleic acid extractions is highly valuable, and may be essential for successful HTS analysis.

## Supporting information

S1 Fig Plots of fragment length distributions for DNA and RNA. (PDF)

S1 Table Values of quantity and quality of DNA and RNA, results of statistical testing, quality metrics for DNA and RNA sequencing and results of gene fusion detection. (XLSX)
