# Supplementary material for: Evaluation of commercial DNA and RNA extraction methods for high-throughput sequencing of FFPE samples
Source: PLoS One. 2018 May 17;13(5):e0197456. doi: 10.1371/journal.pone.0197456 (PMC5957415; doi:10.1371/journal.pone.0197456)

DNA fragment length distributions - SARC1

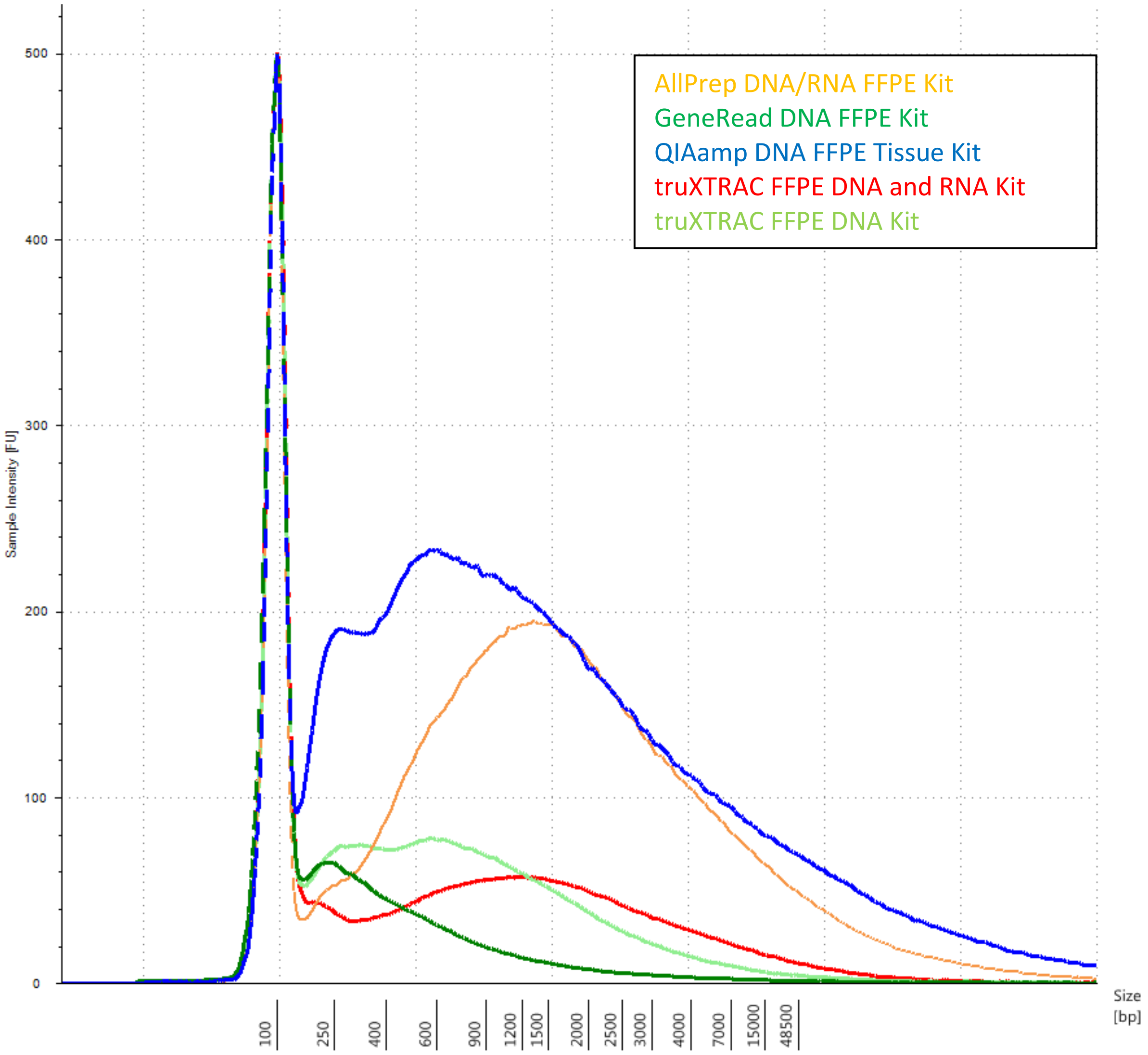

DNA fragment length distributions - SARC2

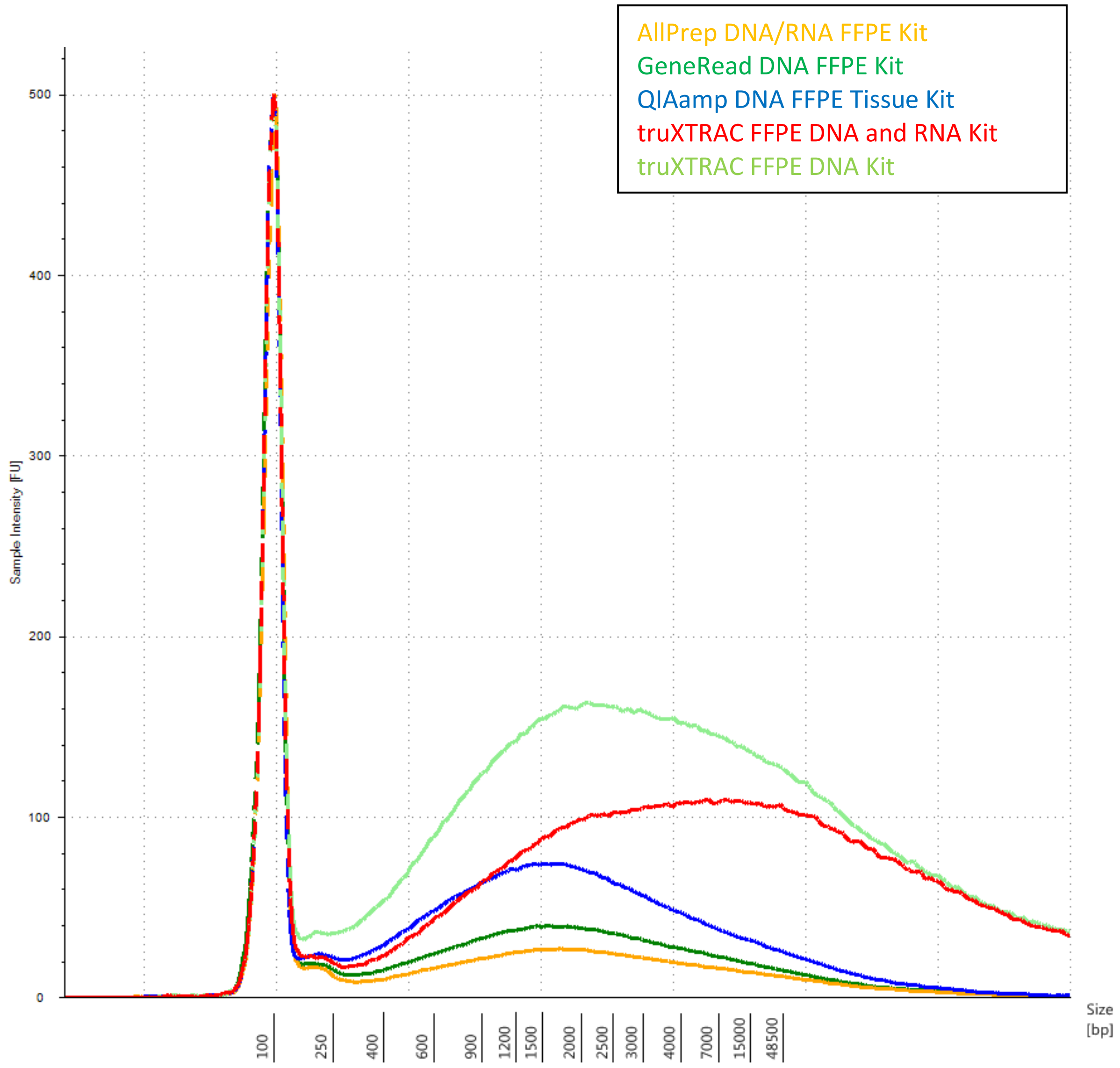

DNA fragment length distributions - SARC3

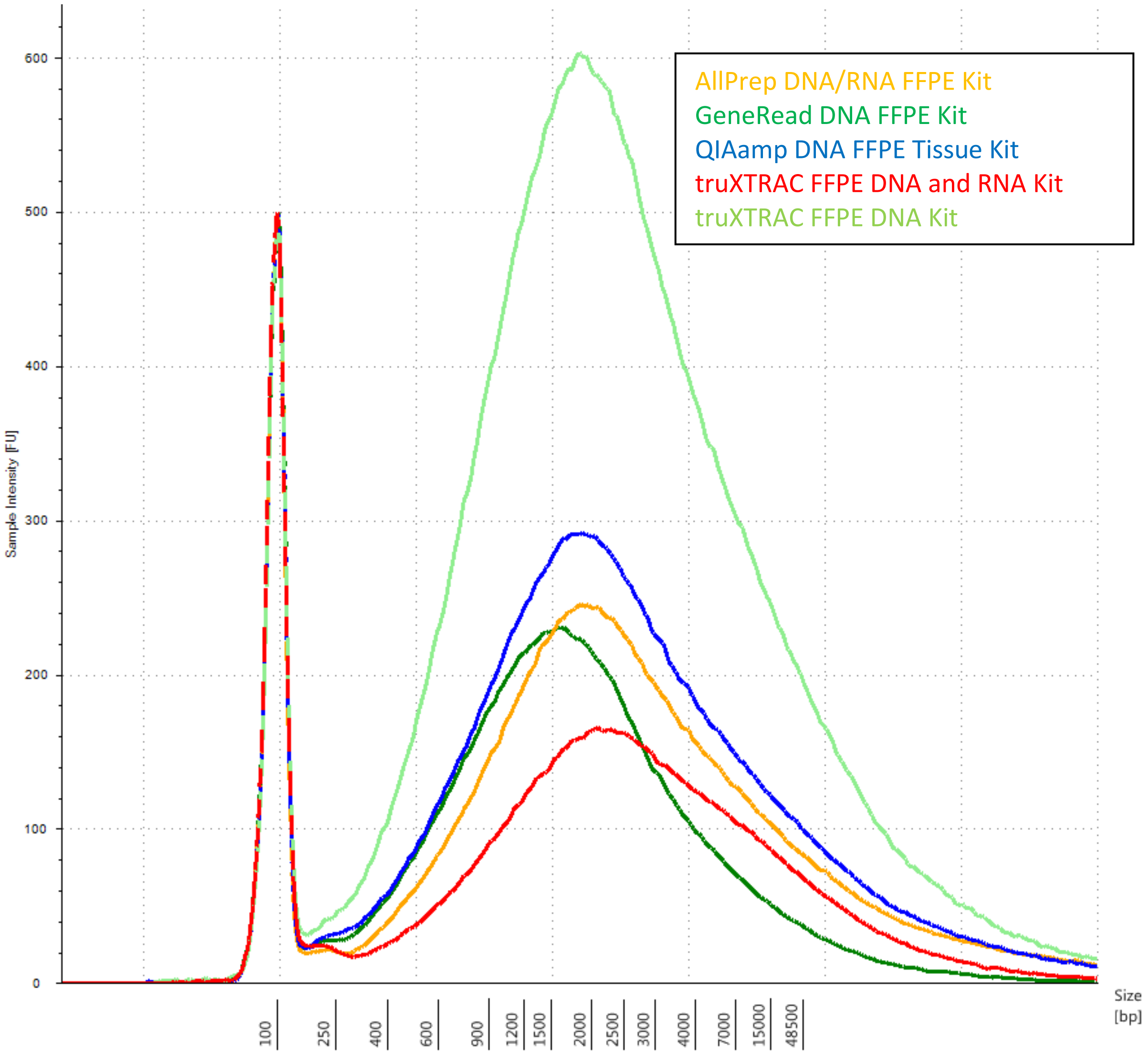

DNA fragment length distributions - SARC4

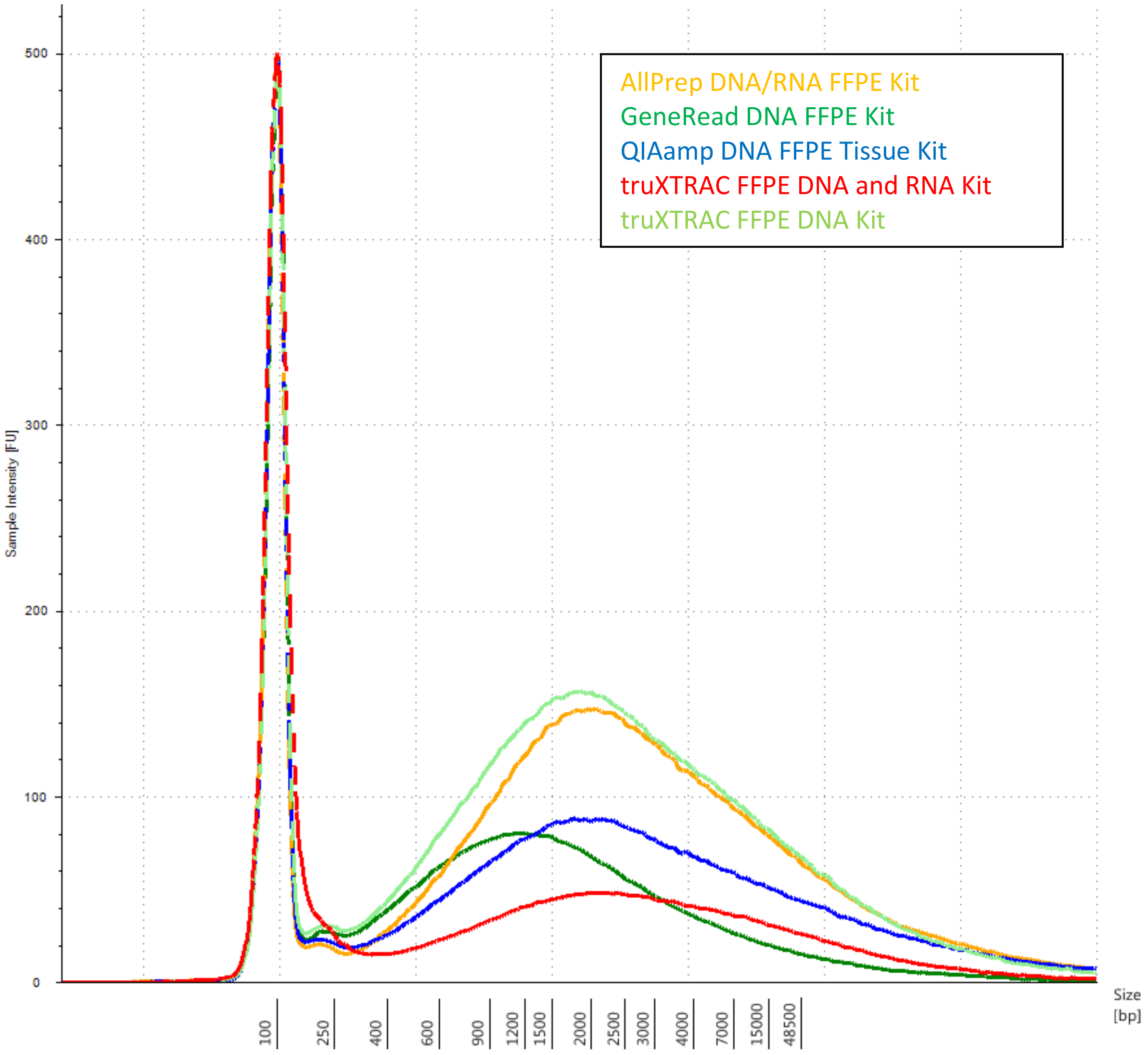

DNA fragment length distributions - SARC5

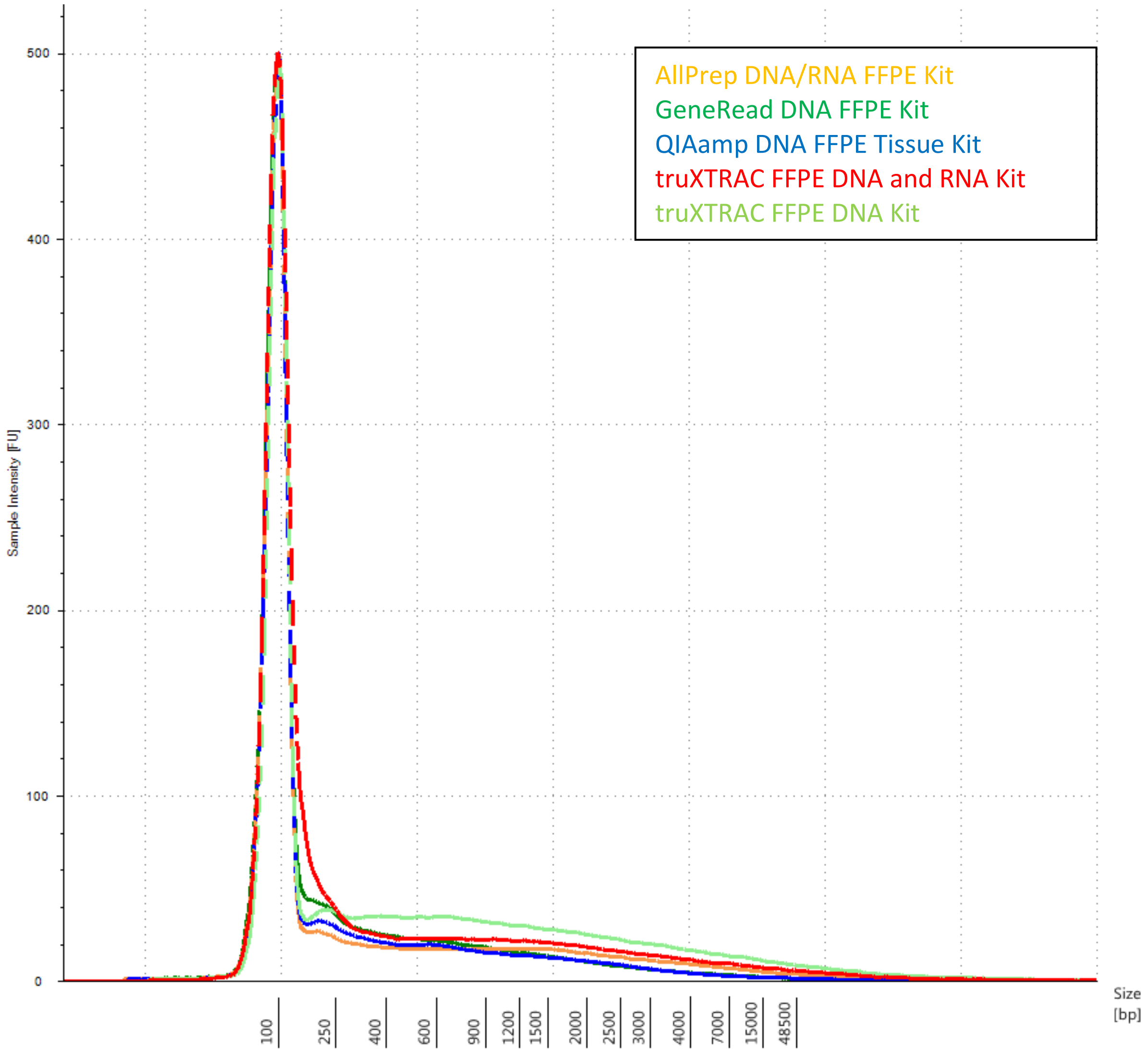

RNA fragment length distributions – SARC1

Electrophoresis File Run Summary

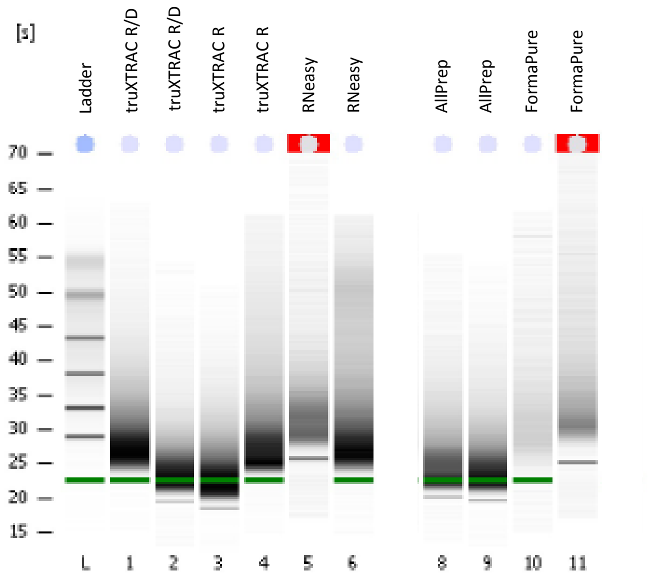

Instrument Information:

Instrument Name: DE20901458 Firmware: C.01.069

Serial#: DE20901458 Type: G2938B

Assay Information:

Assay Origin Path: C:\Program Files\Agilent\2100 bioanalyzer\2100 expert\assays\RNA\Eukaryote Total RNA Nano Series II.xsy

Assay Class: Eukaryote Total RNA Nano

Version: 2.6

Assay Comments: Total RNA Analysis ng sensitivity (Eukaryote)

© Copyright 2003 - 2009 Agilent Technologies, Inc.

Chip Information:

Chip Lot #:

Reagent Kit Lot #:

Chip Comments:

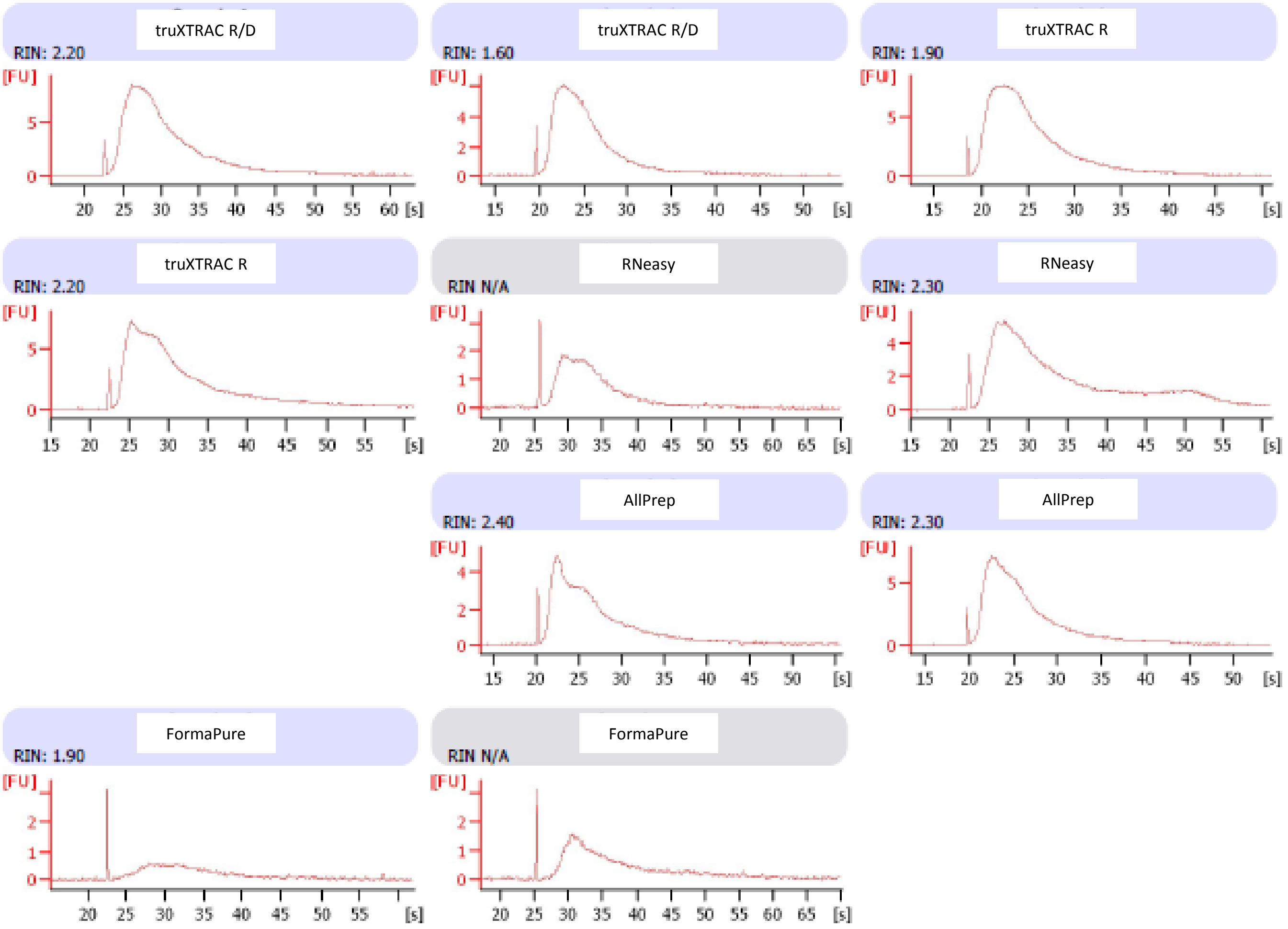

RNA fragment length distributions – SARC2

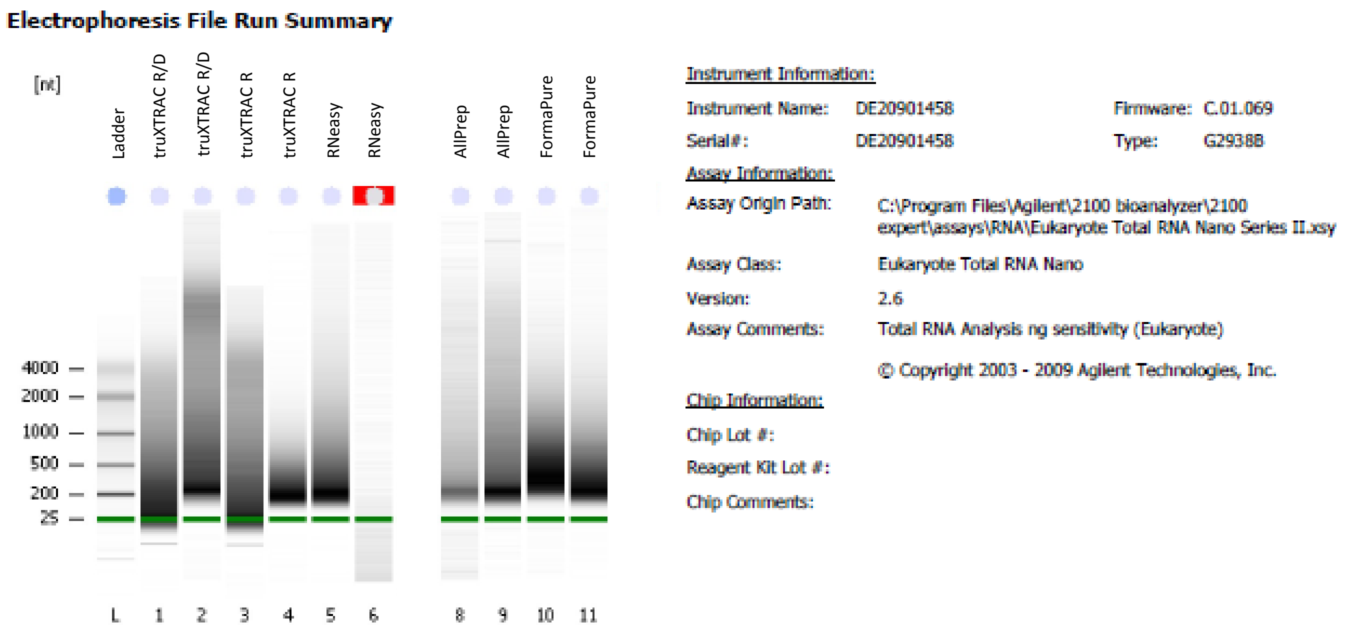

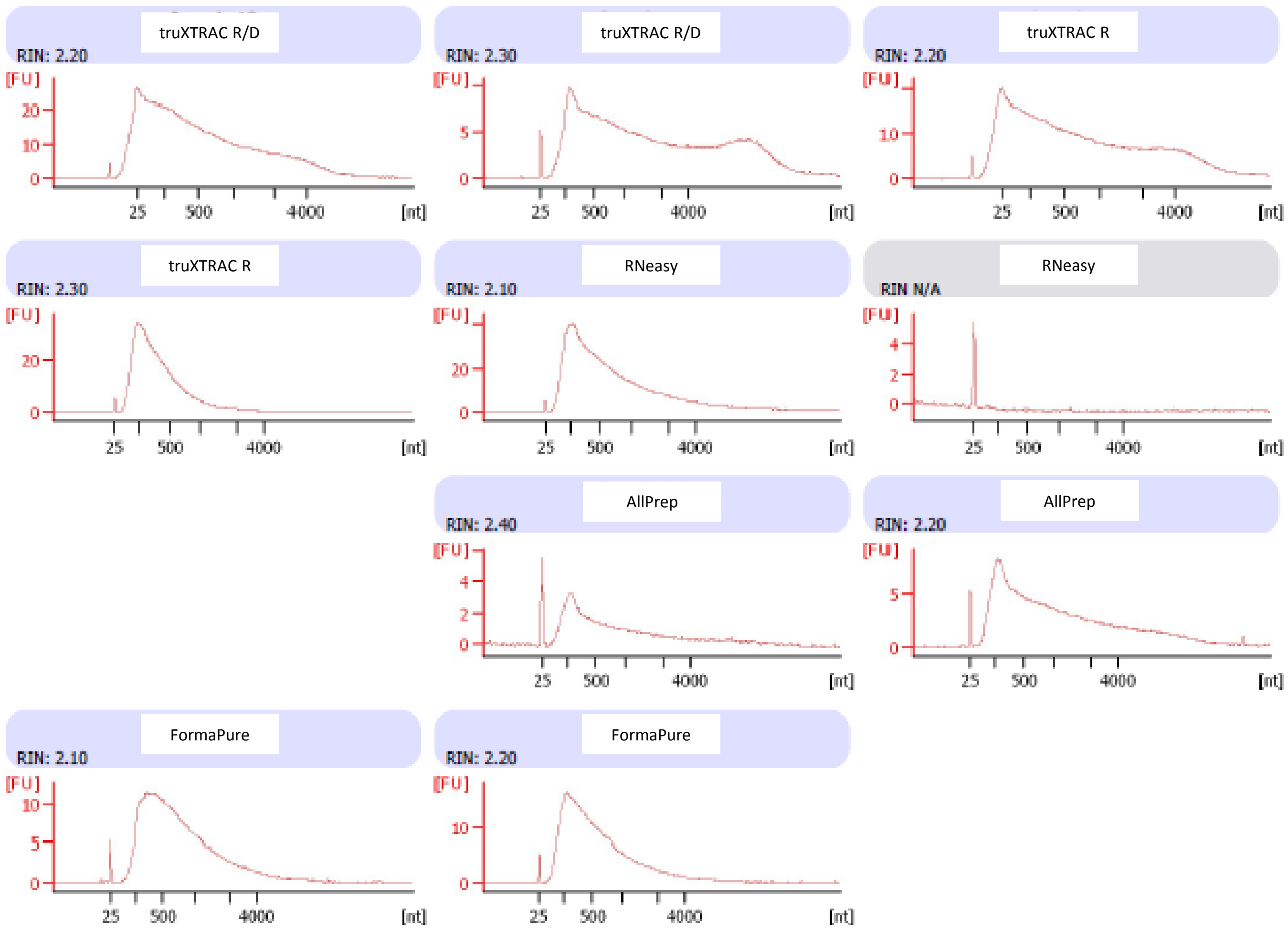

RNA fragment length distributions – SARC3

Electrophoresis File Run Summary

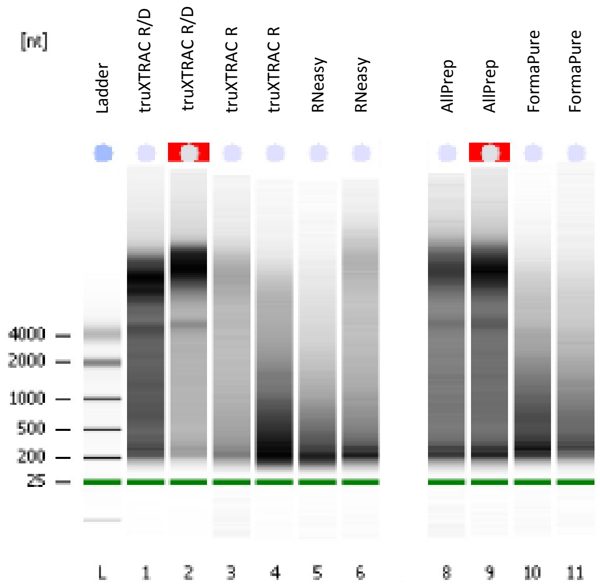

Instrument Information:

Instrument Name: DE20901458

Serial#: DE20901458

Firmware: C.01.069

Type: G2938B

Assay Information:

Assay Origin Path: C:\Program Files\Agilent\2100 bioanalyzer\2100 expert\assays\RNA\Eukaryote Total RNA Nano Series II.xsy

Assay Class: Eukaryote Total RNA Nano

Version: 2.6

Assay Comments: Total RNA Analysis ng sensitivity (Eukaryote)

© Copyright 2003 - 2009 Agilent Technologies, Inc.

Chip Information:

Chip Lot #:

Reagent Kit Lot #:

Chip Comments:

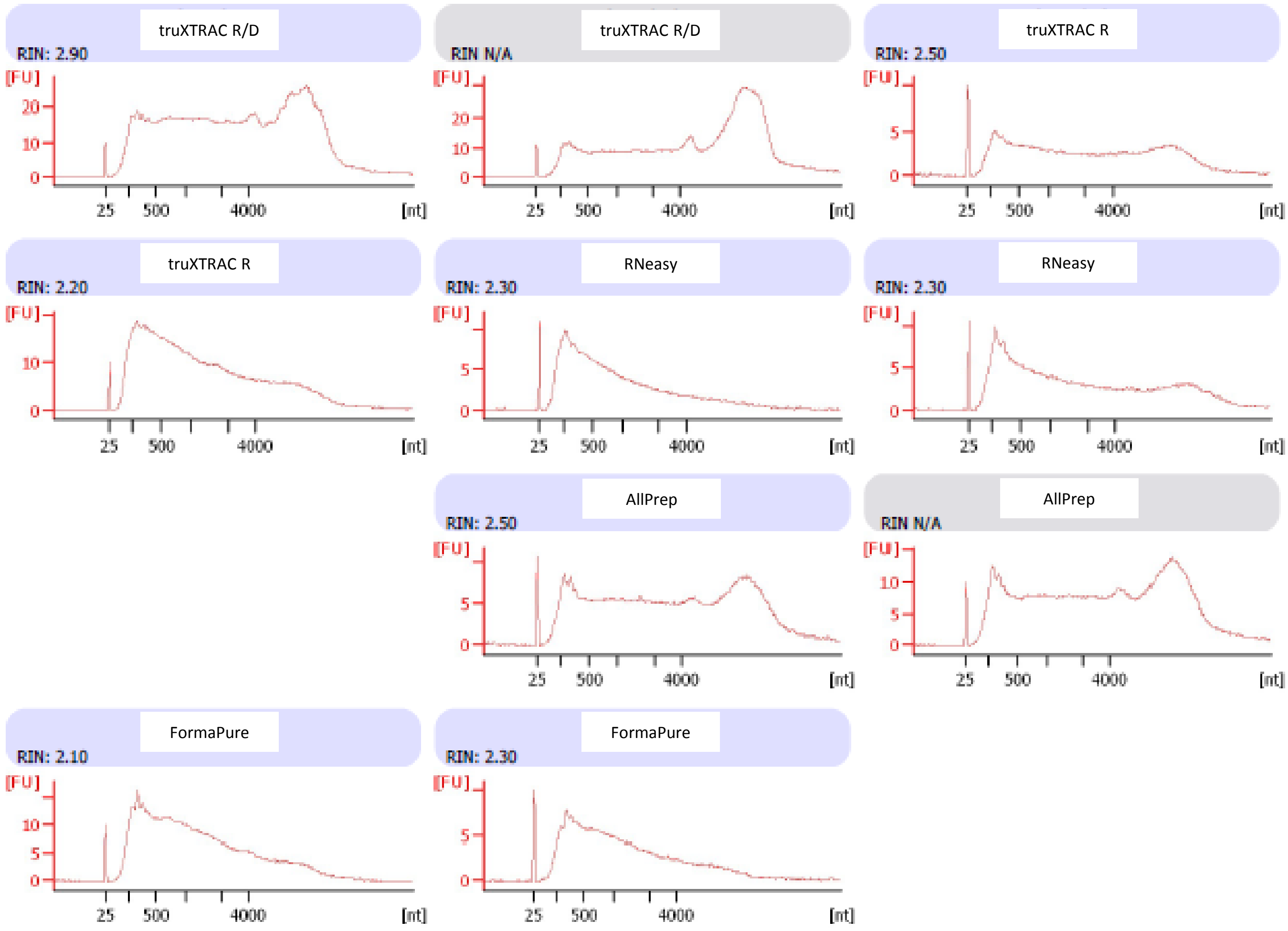

RNA fragment length distributions – SARC4

Electrophoresis File Run Summary

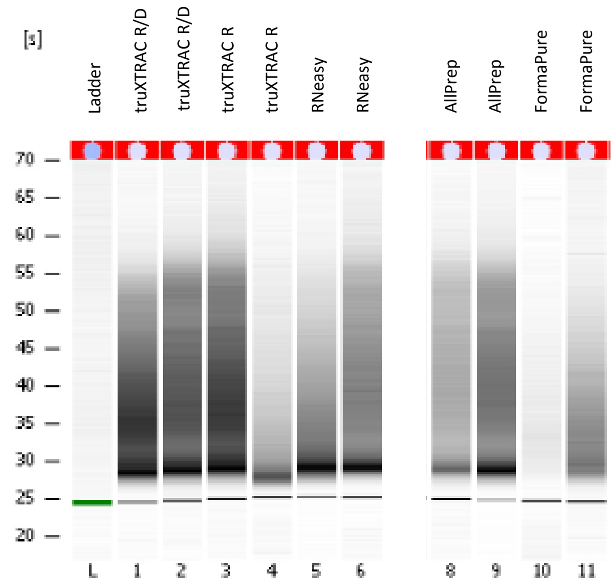

<u>Instrument Information:</u>

Instrument Name:	DE20901458	Firmware:	C.01.069
Serial#:	DE20901458	Type:	G2938B

<u>Assay Information:</u>

Assay Origin Path:	C:\Program Files\Agilent\2100 bioanalyzer\2100 expert\assays\RNA\Eukaryote Total RNA Nano Series II.xsy
Assay Class:	Eukaryote Total RNA Nano
Version:	2.6
Assay Comments:	Total RNA Analysis ng sensitivity (Eukaryote)

© Copyright 2003 - 2009 Agilent Technologies, Inc.

<u>Chip Information:</u>

Chip Lot #:

Reagent Kit Lot #:

Chip Comments:

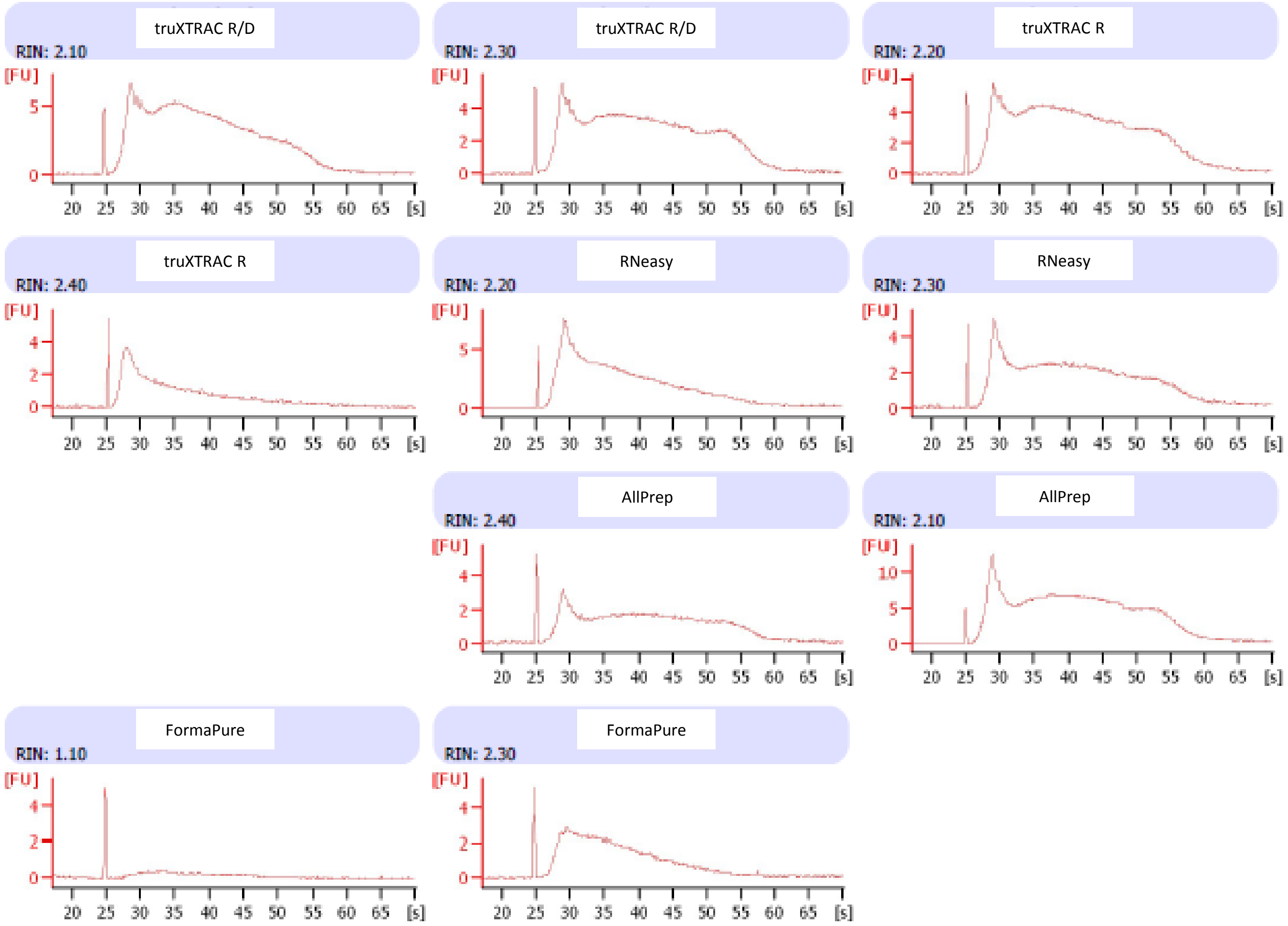

RNA fragment length distributions – SARC5

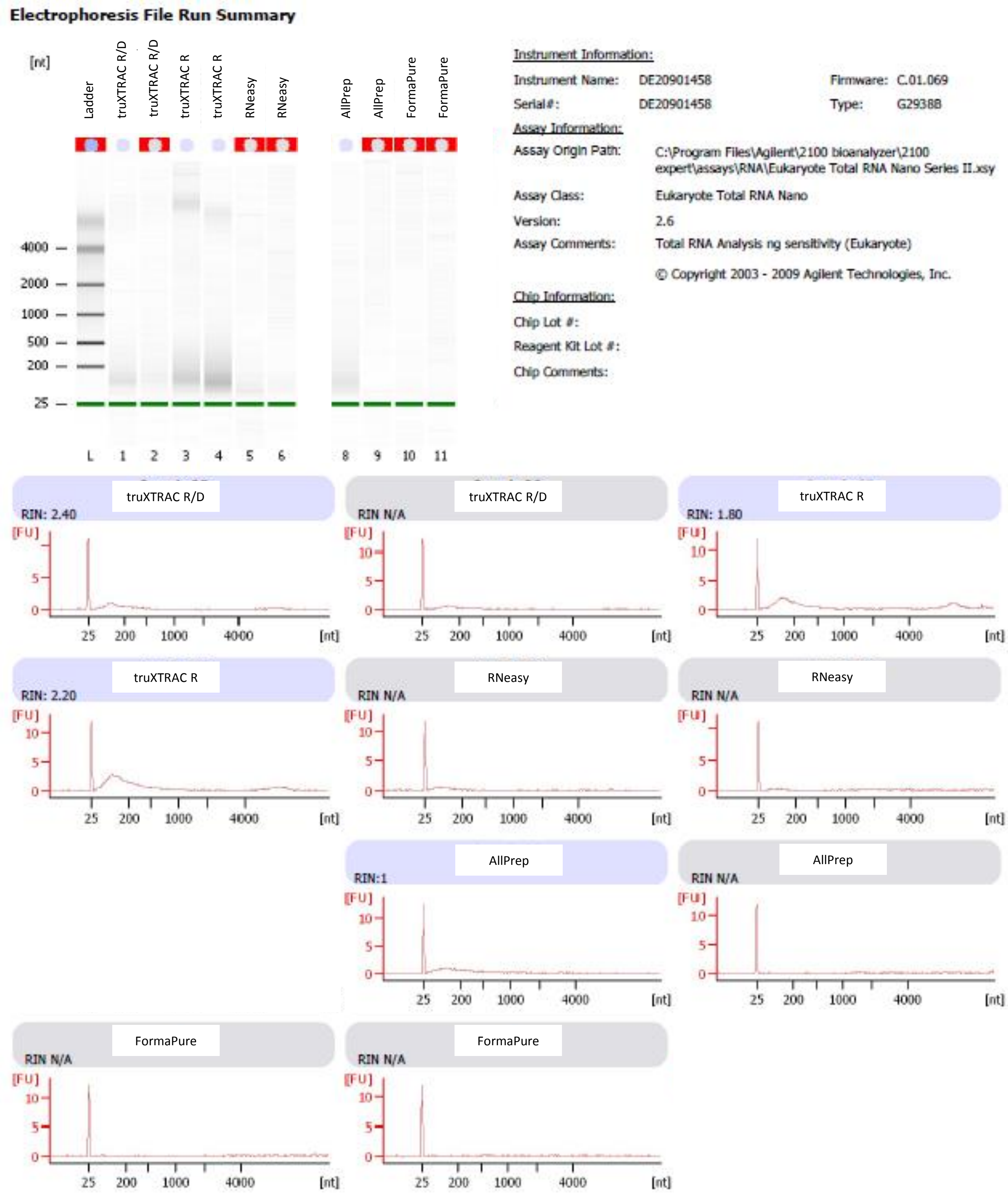

Supplement: S1 Fig — (PDF) [file pone.0197456.s001.pdf]
